# Dr. Barry’s Address

**Published:** 1877-05-20

**Authors:** 


DR. BARRY'S ADDRESS.—We are in receipt of a copy of Dr. W. H. Barry's address before the late meeting of the Arkansas Medical Association, and find it eloquent in phrase, and replete with interesting historical allusions.

